# Supplementary material for: Core functional nodes and sex-specific pathways in human ischaemic and dilated cardiomyopathy
Source: Nat Commun. 2020 Jun 2;11:2843. doi: 10.1038/s41467-020-16584-z (PMC7266817; doi:10.1038/s41467-020-16584-z)
Supplement: Supplementary file 2 — Description of Additional Supplementary Files [file 41467_2020_16584_MOESM2_ESM.docx]

**File Name: Supplementary Data 1.xlsx**

**Description:** Differentially expressed (DE) proteins in ICM *vs* Donor left ventricular myocardium. Estimates were derived using the linear regression model adjusted for gender, where n_ICM_ = 15 and n_donor_ = 15; p-values were adjusted for multiple comparisons by the Benjamini-Hochberg method.

**File Name: Supplementary Data 2.xlsx**

**Description:** DE Proteins in DCM *vs* Donor myocardium. Estimates were derived using the linear regression model adjusted for gender, where n_DCM_ = 14 and n_donor_ = 15; p-values were adjusted for multiple comparisons by the Benjamini-Hochberg method.

**File Name: Supplementary Data 3.xlsx**

**Description:** DE proteins that are common to ICM *vs* Donor and DCM *vs* Donor myocardium. This table is a combination and reorganisation of the Supplementary tables 1 and 2.

**File Name: Supplementary Data 4.xlsx**

**Description:** DE metabolites in ICM *vs* Donor left ventricular myocardium. Estimates were derived using the linear regression model adjusted for gender, where n_ICM_ = 15 and n_donor_ = 15; p-values were adjusted for multiple comparisons by the Benjamini-Hochberg method.

**File Name: Supplementary Data 5.xlsx**

**Description:** DE Proteins in DCM *vs* Donor myocardium. Estimates were derived using the linear regression model adjusted for gender, where n_DCM_ = 14 and n_donor_ = 15; p-values were adjusted for multiple comparisons by the Benjamini-Hochberg method.

**File Name: Supplementary Data 6.xlsx**

**Description:** DE metabolites that are common to ICM *vs* Donor and DCM *vs* Donor myocardium. This table is a combination and reorganisation of the Supplementary tables 4 and 5.

**File Name: Supplementary Data 7.xlsx**

**Description:** Pathway enrichment analysis in ICM *vs* Donor at protein levels. Mean-rank gene set test was used.

**File Name: Supplementary Data 8.xlsx**

**Description:** Pathway enrichment analysis in DCM *vs* Donor at protein levels. Mean-rank gene set test was used.

**File Name: Supplementary Data 9.xlsx**

**Description:** Pathway enrichment analysis in ICM *vs* Donor at metabolite levels. Mean-rank gene set test was used.

**File Name: Supplementary Data 10.xlsx**

**Description:** Pathway enrichment analysis in DCM *vs* Donor at metabolite levels. Mean-rank gene set test was used.

**File Name: Supplementary Data 11.xlsx**

**Description:** Differentially perturbed proteins in each gender in ICM *vs* Donor myocardium. Estimates were derived using the generalised linear model with a gender interaction term, where n_ICM_ = 15 and n_donor_ = 15; p-values were adjusted for multiple comparisons by the Benjamini-Hochberg method.

**File Name: Supplementary Data 12.xlsx**

**Description:** Differentially perturbed proteins in each gender in DCM *vs* Donor myocardium. Estimates were derived using the generalised linear model with a gender interaction term, where n_DCM_ = 14 and n_donor_ = 15; p-values were adjusted for multiple comparisons by the Benjamini-Hochberg method.

**File Name: Supplementary Data 13.xlsx**

**Description:** Differentially perturbed metabolites in each gender in ICM *vs* Donor myocardium. Estimates were derived using the generalised linear model with a gender interaction term, where n_ICM_ = 15 and n_donor_ = 15; p-values were adjusted for multiple comparisons by the Benjamini-Hochberg method.

**File Name: Supplementary Data 14.xlsx**

**Description:** Differentially perturbed metabolites in each gender in DCM *vs* Donor myocardium. Estimates were derived using the generalised linear model with a gender interaction term, where n_DCM_ = 14 and n_donor_ = 15; p-values were adjusted for multiple comparisons by the Benjamini-Hochberg method.

**File Name: Supplementary Data 15.xlsx**

**Description:** Instrument Settings Metabolites
