# Supplementary material for: Core functional nodes and sex-specific pathways in human ischaemic and dilated cardiomyopathy
Source: Nat Commun. 2020 Jun 2;11:2843. doi: 10.1038/s41467-020-16584-z (PMC7266817; doi:10.1038/s41467-020-16584-z)
Supplement: Supplementary file 18 — Reporting Summary [file 41467_2020_16584_MOESM18_ESM.pdf]

nature research

Corresponding author(s): John O'Sullivan

Last updated by author(s): Mar 30, 2020

Reporting Summary

Nature Research wishes to improve the reproducibility of the work that we publish. This form provides structure for consistency and transparency in reporting. For further information on Nature Research policies, see Authors & Referees and the Editorial Policy Checklist .

Statistics

For all statistical analyses, confirm that the following items are present in the figure legend, table legend, main text, or Methods section.

n/a	Confirmed	
☐	☒	The exact sample size (*n*) for each experimental group/condition, given as a discrete number and unit of measurement
☐	☒	A statement on whether measurements were taken from distinct samples or whether the same sample was measured repeatedly
☐	☒	The statistical test(s) used AND whether they are one- or two-sided<br>*Only common tests should be described solely by name; describe more complex techniques in the Methods section.*
☐	☒	A description of all covariates tested
☐	☒	A description of any assumptions or corrections, such as tests of normality and adjustment for multiple comparisons
☐	☒	A full description of the statistical parameters including central tendency (e.g. means) or other basic estimates (e.g. regression coefficient) AND variation (e.g. standard deviation) or associated estimates of uncertainty (e.g. confidence intervals)
☐	☒	For null hypothesis testing, the test statistic (e.g. *F*, *t*, *r*) with confidence intervals, effect sizes, degrees of freedom and *P* value noted<br>*Give P values as exact values whenever suitable.*
☒	☐	For Bayesian analysis, information on the choice of priors and Markov chain Monte Carlo settings
☒	☐	For hierarchical and complex designs, identification of the appropriate level for tests and full reporting of outcomes
☐	☒	Estimates of effect sizes (e.g. Cohen's *d*, Pearson's *r*), indicating how they were calculated

Our web collection on statistics for biologists contains articles on many of the points above.

Software and code

Policy information about availability of computer code

Data collection	For proteomics, DDA data were processed with Andromeda in MaxQuant v1.6.0.9, and the MaxQuant output were used to generate a spectral library in Spectronaunt v12. For metabolomics, the analysis software MultiQuant 3.0 (ABSciex) was used for MRM Q1/Q3 peak integration of the raw data files (Analyst software, v.1.6.2; ABSciex).
Data analysis	All statistical analyses were done in R(3.5.2), using lmFit and eBayes functions implemented in the limma R package. Pathway analyses were performed with mean-rank gene set tests, using the geneSetTest function from limma, where t-statistics from the corresponding differential expression analysis were ranked. Pathways were annotated by the Kyoto Encyclopedia of Genes and Genomes (https://www.kegg.jp). Pathway visualisations were performed using the pathview R package. Network plots of DE proteins and metabolites were generated with the igraph R package.

For manuscripts utilizing custom algorithms or software that are central to the research but not yet described in published literature, software must be made available to editors/reviewers. We strongly encourage code deposition in a community repository (e.g. GitHub). See the Nature Research guidelines for submitting code & software for further information.

Data

Policy information about availability of data

All manuscripts must include a data availability statement. This statement should provide the following information, where applicable:

- Accession codes, unique identifiers, or web links for publicly available datasets
- A list of figures that have associated raw data
- A description of any restrictions on data availability

The mass spectrometry metabolomics data have been deposited to Metabolomics workbench with DataTrack IDs 1850, 1861, 1862, 1865 (username: efep20; password: IHDdcm2019). The mass spectrometry proteomics data have been deposited to the ProteomeXchange Consortium via the PRIDE partner repository with the dataset identifier PXD016617 (username: reviewer17062@ebi.ac.uk; password: DMOONI9l). There are no restrictions on data availability.

Field-specific reporting

Please select the one below that is the best fit for your research. If you are not sure, read the appropriate sections before making your selection.

☒ Life sciences ☐ Behavioural & social sciences ☐ Ecological, evolutionary & environmental sciences

For a reference copy of the document with all sections, see nature.com/documents/nr-reporting-summary-flat.pdf

Life sciences study design

All studies must disclose on these points even when the disclosure is negative.

Sample size	Sample sizes were determined by a preliminary study that determined the effect size, d, of commonly-reported differential proteins in heart failure (e.g. collagen, TGF-beta) to be 3.5-4.0, requiring 10 hearts per group to give 95% to determine a difference between groups. Therefore, we used 15 hearts per group to ensure adequate power.
Data exclusions	No samples were excluded.
Replication	As samples were precious, we did not replicate the results. However, we previously replicated metabolomic data in similar human heart samples in two independent runs (Cao...O'Sullivan, Eur J Heart Failure, 2019). Furthermore, we replicated previously-reported differential proteins in human heart failure in this study, as detailed in the manuscript.
Randomization	The sample run order for mass spectrometry was randomized across pathological grouping, age, gender, and BMI.
Blinding	All analyses of the data was performed in a blinded fashion by the statistical team. For PCA, heatmap, and network analyses the group assignments were added AFTER the plots were generated.

Reporting for specific materials, systems and methods

We require information from authors about some types of materials, experimental systems and methods used in many studies. Here, indicate whether each material, system or method listed is relevant to your study. If you are not sure if a list item applies to your research, read the appropriate section before selecting a response.

Materials & experimental systems

n/a	Involved in the study	
☒	☐	Antibodies
☒	☐	Eukaryotic cell lines
☒	☐	Palaeontology
☒	☐	Animals and other organisms
☐	☒	Human research participants
☒	☐	Clinical data

Methods

n/a	Involved in the study	
☒	☐	ChIP-seq
☒	☐	Flow cytometry
☒	☐	MRI-based neuroimaging

Human research participants

Policy information about studies involving human research participants

Population characteristics	Both types of heart failure have relevant covariate characteristics. Particularly important are age and gender. Therefore, we compared hearts by matching for age (the median age of each condition group are as follows: ICM = 54, DCM = 56 and donors = 54) and gender (the gender distribution of each condition group: in ICM, female = 5 and male = 10; in DCM, female = 5 and male = 9; in donors, female = 8 and male = 7). Ordinarily, BMI is also an important covariate in metabolism and cardiovascular disease. However, in advanced heart failure, due to cardiac cachexia and sarcopenia, BMI is an unreliable covariate and cannot be adjusted for in a standard fashion.
Recruitment	Participants were not recruited per se. We examined samples from patients with the two most common types of heart failure and cardiac transplantation, and uniquely, age and gender-matched histopathologically pre-mortem normal hearts procured from heart transplant donors whose hearts were not used for logistical reasons.
Ethics oversight	The study was approved by the Ethics Committee of The University of Sydney (USYD #2016/923) and was conducted in accordance with the Declaration of Helsinki.

Note that full information on the approval of the study protocol must also be provided in the manuscript.
